# Molecular Simulation of the Adsorption and Diffusion in Cylindrical Nanopores: Effect of Shape and Fluid–Solid Interactions

**DOI:** 10.3390/molecules24030608

**Published:** 2019-02-09

**Authors:** Harry Cárdenas, Erich A. Müller

**Affiliations:** Department of Chemical Engineering, Imperial College London, South Kensington Campus, London SW7 2AZ, UK.; h.cardenas15@imperial.ac.uk

**Keywords:** adsorption, diffusion, molecular dynamics, nanopores

## Abstract

We report on molecular simulations of model fluids composed of three tangentially bonded Lennard-Jones interaction sites with three distinct morphologies: a flexible “pearl-necklace” chain, a rigid “stiff” linear configuration, and an equilateral rigid triangular ring. The adsorption of these three models in cylindrical pores of diameters 1, 2, and 3 nm and with varying solid–fluid strength was determined by direct molecular dynamics simulations, where a sample pore was placed in contact with a bulk fluid. Adsorption isotherms of Type I, V, and H1 were obtained depending on the choice of pore size and solid–fluid strength. Additionally, the bulk-phase equilibria, the nematic order parameter of the adsorbed phase, and the self-diffusion coefficient in the direction of the pore axis were examined. It was found that both the molecular shape and the surface attractions play a decisive role in the shape of the adsorption isotherm. In general, the ring molecules showed a larger adsorption, while the fully flexible model showed the smallest adsorption. Morphology and surface strength were found to have a lesser effect on the diffusion of the molecules. An exceptional high adsorption and diffusion, suggesting an enhanced permeability, was observed for the linear stiff molecules in ultraconfinement, which was ascribed to a phase transition of the adsorbed fluid into a nematic liquid crystal.

## 1. Introduction

Nanoporous materials have become a common staple in modern membrane separation, catalysis, and biological and adsorption engineering. At this level of confinement, it is the energetical and morphological molecular-level interactions that dominate the observed macroscopic behavior, as opposed to the more classical macroscopic fluid descriptors, such as viscosity and pressure, whose sheer definition breaks down in this highly anisotropic nanodomain. The ultraconfinement of fluids, along with the balance between fluid–fluid and solid–fluid interactions, is responsible for providing many unusual physical phenomena, such as ultrahigh local pressures that can act as catalyzers for chemical reactions [1], the rapid (or slowdown of) transport through nanoporous carbon structures, unexpected selectivities upon permeation [2], and anomalous transport in crowded biological media [3], to name but a few. Moreover, confinement can affect the fluid properties and induce unique phase changes and structural transitions not seen or anticipated in the corresponding bulk fluids [4]. While experiments provide for the discovery of intriguing structure–dynamics relationships, the challenges associated with the characterization of the porous media, as well as the difficulties in measuring both the adsorption and diffusivities in nanoscopic environments [5], hamper the interpretation of the results and the formulation of comprehensive theories to describe the underlying physics. Furthermore, a fundamental understanding of the molecular processes is a requisite for the design of new materials that make full use of the new opportunities that arise from the deployment of fluids in nanoconfined spaces. Molecular simulation is well posed to explore these effects.

It is well known that the structure and energy of the pore walls control the shape of adsorption isotherms, leading to the six different types of isotherms reviewed in the recent International Union of Pure and Applied Chemistry (IUPAC) classification [6]. An important feature of the pore structure is the geometry as it directly influences the interactions between the particles and the walls. Monson [7] presented a review detailing molecular simulation results of adsorption and desorption of a Lennard-Jones (LJ) 12–6 fluid in different pore geometries, i.e., slit pore, closed end, wedge, and ink-bottle pore, relating the effect of the geometry on the hysteresis phenomena. Their results rationalize how the geometry of the pore has a substantial impact on the shape of the adsorption isotherm [8]. Many experimental data confirm these simulation results [9,10,11]. Nevertheless, when considering adsorption, besides the structure of the pore, the morphology of the fluid molecules (i.e., their overall shape) is equally relevant as the unique peculiarities of the molecular configuration may lead to different adsorption behavior. A well-documented example is the opposing adsorption of linear and branched alkanes adsorbing in narrow-window zeolites, where molecular sieving affects the adsorption and diffusion, providing for effective shape selectivity [12]. Smit et al. [13,14] explored the adsorption of C6 hydrocarbons for different molecular configurations, i.e., linear (n−C6) and branched (3-methylpentane and 2,2-dimethylbutane), in several nanoporous zeolites. In silicalite (MFI), dibranched alkanes had difficulty penetrating the nanopores as they were bulkier (in the 3-D sense) than the monobranched and linear alkane counterparts. This configurational entropy effect caused the molecular loading of hexane isomers to follow a distinct sorption hierarchy of linear > monobranched > dibranched. When another type of zeolite (AFI) with cylindrical channels of 0.73 nm radius was studied, the sorption hierarchy was inverted, i.e., linear < monobranched < dibranched, as the channels in AFI were large enough to accommodate the bulky dibranched molecule. A similarly natured work was published by Jiang and Sandler [15], showcasing the different adsorption behaviors for some hydrocarbons, in particular the difference between C5 isomers. Following the same ideas, Sarkisov et al. [16] used Grand Canonical Monte Carlo and molecular dynamics to simulate the adsorption of methane, n−alkanes, cyclohexane, and benzene in two different nanoporous materials: bipyridine molecular squares and isoreticular metal–organic frameworks (IRMOFs). A relevant outcome of that study was the comparison of the adsorption isotherms of two C6 isomers, n−hexane and cyclohexane, in pores where there were no size exclusion (sieving) effects. Here, it could be seen that the adsorbed amount for the ring-like isomer was larger than that for the linear one.

A parallel and equally relevant aspect for separation processes involves the kinetics, governed by the diffusion and transport of the adsorbed molecules inside the pores. From an experimental point of view, it is challenging to measure diffusion coefficients of confined fluids with accuracy [17,18]. Molecular simulation, on the other hand, provides an unequivocal means of calculating transport coefficients, although the sparsity of molecules produced by the confinement can lead to poor statistics and hence large uncertainties. As with the case of adsorption, much effort has been placed on studying the effects of subtleties of the pore morphology on the diffusion [19]. Notable exceptions are focused on some key industrial separations, such as differences in the diffusion of alkane isomers in zeolites, where linear alkanes are seen to diffuse faster than their branched isomers [20].

**Figure 1:**
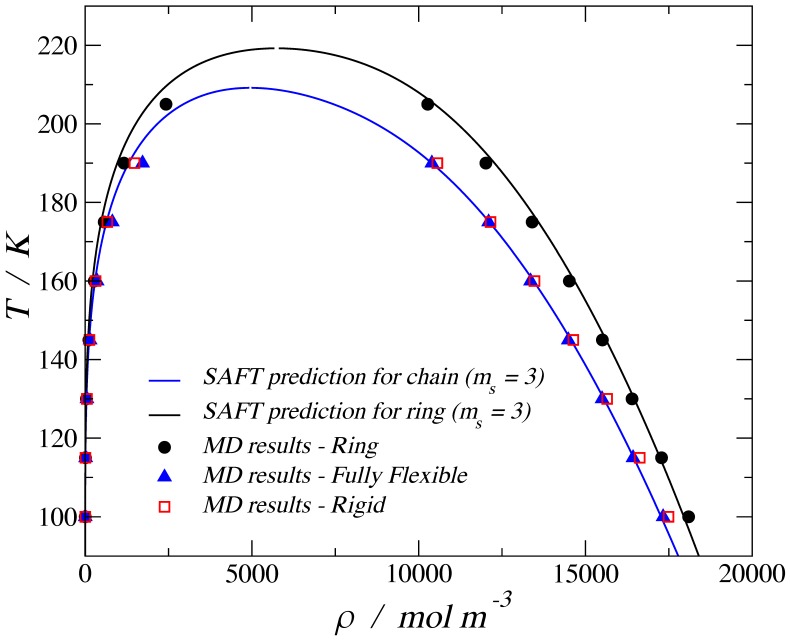
Temperature–density vapor–liquid coexistence curve for a 3-bead Lennard-Jones fluid. The solid blue line corresponds to the SAFT-VR-Mie equation of state (EoS) for chains, the solid black line corresponds to the SAFT-VR Mie EoS for rings [24], the filled black circles are obtained by molecular dynamic (MD) simulations for the ring trimer, the filled blue triangles are the simulation results for a fully flexible trimer, and the empty red squares are the simulation results for a rigid (stiff) linear trimer.

The abridged selection of examples above suggests that the molecular shape must play a role on the adsorption and transport of molecules within nanoporous materials. A key attribute that defines thermodynamic behavior in the fluid state is the shape of the molecules. The effect of chain length and flexibility on bulk fluid behavior is well known experimentally and reproduced in molecular simulations [21]. For the case of three tangent and identical isotropic beads, henceforth called trimers, it is known that fully flexible chains and their rigid linear counterparts exhibit the same vapor–liquid equilibria (VLE) behavior. However, when the number of segments, *ms*, is increased, the rigid configurations are able to reach a higher density than the flexible chains [22] and, for given state points, are capable of exhibiting a nematic liquid crystal phase [23]. Another molecular morphology of interest is the ring-like or discotic configuration, for which molecular simulation and theoretical studies are available [24]. When the flexible chain and the ring-like configuration are compared, the latter gives a higher critical point and larger values of density than the flexible chain. Figure 1 shows the VLE for LJ chains using three morphologies, where the symbols correspond to molecular dynamic (MD) simulations, and the solid lines are the predictions obtained using the variable range Mie statistical associating fluid theory (SAFT-VR-Mie) equation of state (EoS). For the case of trimers, the corresponding SAFT theory for chains [25] gives the same VLE predictions for both the fully flexible and the rigid linear configuration, and this agrees with the MD results. For the case of planar ring compounds, higher liquid densities and critical points are predicted by the theory [24] and confirmed by molecular simulation results.

This work aims to study systematically through MD simulations how the molecular morphology affects the adsorption of model trimers into cylindrical nanopores. Experimental results suggest that the VLE properties of fluids under extreme confinement are different from those in the bulk phase [26], and it is expected that similar differences exist in terms of the adsorption and diffusion in confinement. We employed three different pore sizes and three distinct fluid–solid energy interaction values to test three molecular configurations of trimers: flexible chain, rigid (stiff) linear chain, and ring (triangle). A representation drawn to scale is shown in Figure 3, where the three different morphologies are presented next to the three pore sizes used in this work. We employed generic models composed of linked spherical beads as they are successfully used as molecular models for coarse-grained representation of fluids. While no attempt was made here to link these models to experimental properties, tangent Mie models, of which the LJ potential is a particular case, have been successfully used as coarse-grained analogues for the representation of a wide range of fluids [27], appropriately parametrized [28], and have been successful in describing volumetric [29], transport [30], and interfacial [31] properties of bulk and confined fluids [32]. For example, a three-tangent bead model has been proposed as a model for n-nonane [33], while a trimer in the form of a triangle is a suitable model for benzene [34].

## 2. Simulation Details

**Figure 2:**
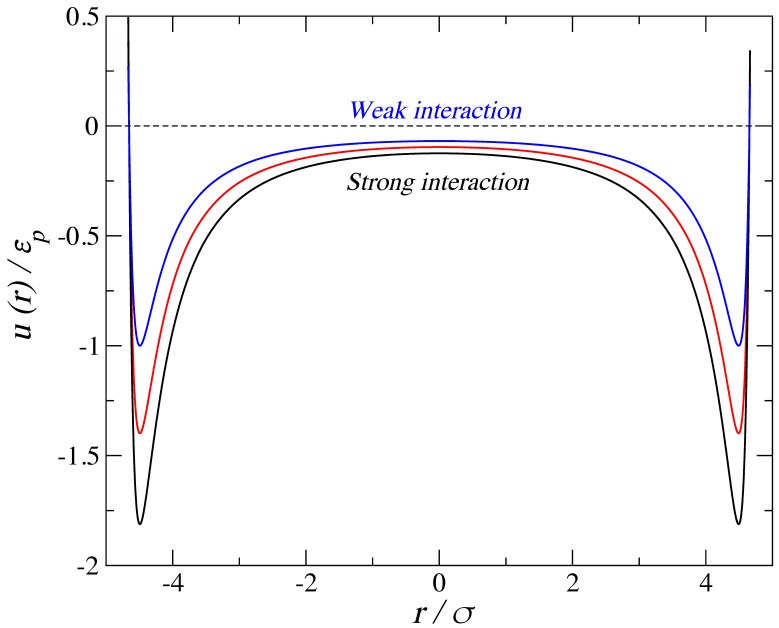
Fluid–solid intermolecular potentials for the cylindrical pore of radius 1.5 nm using three different values of fluid-wall energy, normalized with respect to the well depth of the weakest (*ε*_p_ = 13.8 *k*_b_). Distances are normalized with respect to the soft diameter of a single fluid bead (*σ* = 0.3 nm).

The interaction between the fluid particles and the solid wall of the nanopore were modeled through the Mie potential, $u_{Mie}(r)$, a generalized form of the LJ potential:

(1) $$u_{Mie}(r)=\frac{n}{n-m}{\left(\frac{n}{m}\right)}^{m/(n-m)}\varepsilon \left[{\left(\frac{\sigma }{r}\right)}^{n}-{\left(\frac{\sigma }{r}\right)}^{m}\right]$$

where *σ* is a length scale that corresponds loosely with an effective segment diameter, *r* is the distance between the centers of the particles, *n* and *m* are the repulsive and attractive exponents for the potential, respectively, and *ε* is the energy scale corresponding to the potential well depth. The interactions between the fluid molecules are represented by the LJ potential (*n* = 12 and *m* = 6). Figure 2 shows the relative shapes of the solid–fluid potential for the cylindrical wall for three different values of fluid-wall energy, *ε*p, for the largest pore considered. The strong interaction is labeled C_cylinder,1_−LJ_fluid_, the medium interaction is C_cylinder,2_−LJ_fluid_, and the weak interaction is C_cylinder,3_−LJ_fluid_, with the interaction parameters detailed in Table 1. An arbitrary choice was made with respect to the absolute values of these size and energy parameters in order to provide measurable quantities. All results may be generalized by taking into account reduced properties; throughout the paper, we have expressed these reduced units alongside the absolute ones. Similarly, an arbitrary mass of 100 g·mol^−1^ was assigned to each Mie sphere.

The molecular fluid models used for this work were built by joining three tangential segments to form the trimers. The bond stretching between two adjacent covalent-like bonded atoms *i* and *j* is represented by a harmonic potential:

(2) $$u_{b}(r_{ij})=\frac{1}{2}k_{12}{\left(r_{ij}-r_{0}\right)}^{2}$$

where *k*_12_ is the harmonic force constant in kJ·mol^−1^·m^−2^, and *r*_0_ is the equilibrium bond length set here to be equal to the value of *σ* of the fluid. The force constant chosen for this harmonic potential had a value of 6000 kJ·mol^−1^·m^−2^. This is a relatively large value, essentially a rigid spring maintaining the bead tangent. A harmonic angle potential was employed to moderate the rigidity of linear chains:

(3) $$u_{a}(r_{ijk})=\frac{1}{2}k_{123}{\left(\theta -\theta_{0}\right)}^{2}$$

where *θ* is the angle between the bonds connecting the three segments of the chain, *k*_123_ is the force constant in kJ·mol^−1^·rad^−2^, and *θ*_0_ is the reference angle, taken to be 180°. The fully flexible model is defined as a chain with *k*_123_ = 0, while the rigid linear is characterized by *k*_123_ = 3000 kJ·mol^−1^·rad^−2^. The ring trimer is defined by an equilateral triangle of side *σ* formed by the centers of the three molecules.

**Figure 3:**
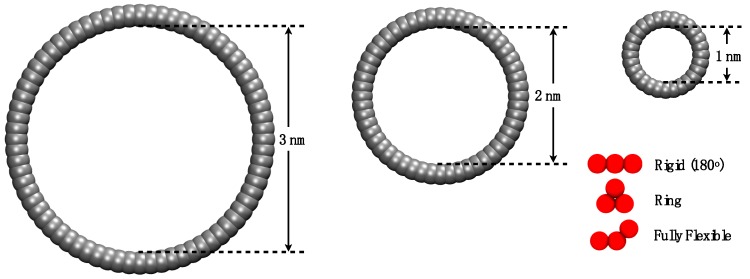
Representation of the pores and molecules employed, drawn to scale. Diagram shows the convention employed to define the inner pore diameter.

**Figure 4:**
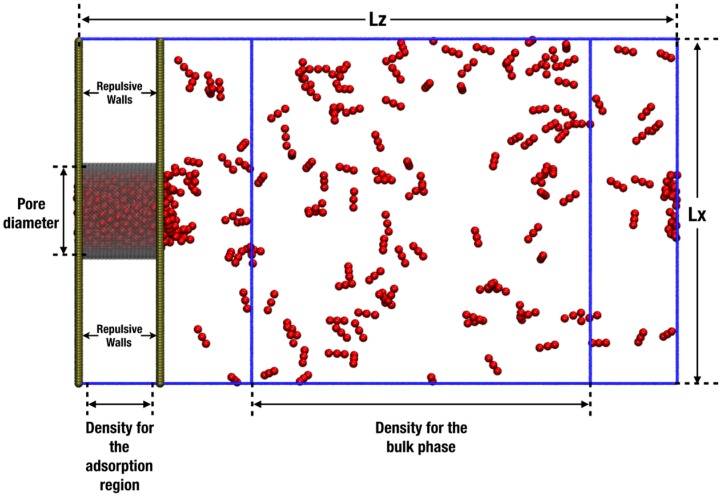
Schematic representation of the simulation box used to obtain the adsorption isotherms and the bulk density. Periodic boundary conditions are applied in all Cartesian directions.

The porous material used in this work was a cylindrical single-walled nanopore with a simple cubic lattice structure. Wall sites were spheres (*σ* = 0.2 nm) fused at a bond distance of 0.13 nm, guaranteeing a rather smooth surface. The length of the cylinder in the *z* direction was 3 nm, and we employed three different pore radius values, as shown in Figure 3: 0.5 nm, 1.0 nm, and 1.5 nm (from right to left). The choice of sizes was made to explore both the ultraconfined pores, where the walls exert influence on the adsorbed fluid (pores of less than 2 nm), and those where some “bulk-like” fluid will be expected in the central region [35]. A schematic of the simulation cell is displayed in Figure 4, where the simulation box contained both the confined phase and the bulk phase in contact. Two purely repulsive [36] walls were placed in the box on the plane of the entrance to the pores to avoid access to the external area of the pore. The dimensions of the simulation box depended on the pore radius *r*p; *L*x = *L*y = (2*r*p + 9.4) nm and *L*z = (1.5*L*x + 3.4) nm. The averages to calculate the bulk density and the adsorbed density were taken far from the interface (at 3*σ* and 10*σ*, respectively) to probe a homogeneous phase, as shown in Figure 4.

We used the GROMACS 5.1 simulation open source suite [37] to perform the MD simulations. The simulation cell had periodic boundary conditions in all three Cartesian directions. The cut-off radius for all the potentials was 1.5 nm (~5*σ*). All the adsorption simulations were carried out in the Canonical Ensemble (constant number of particles, *N*; total volume, *V*; and temperature, *T*). The temperature was kept constant at 150 K (*k*_b_*T*/*ε* = 1.5), which was below the bulk critical temperature of both the chain and the ring-like configuration by a Nosé–Hoover thermostat, using a relaxation constant of 1 ps. The number of beads used for both the cylindrical pore and the repulsive wall were close to 30,000 (depending on the pore), while the number of beads for the fluid phase was approximately 2000. All the simulations were performed with a 0.008 ps time step and a total production run length (after equilibration) of 70 ns.

The pore diameter is defined as the distance between opposing walls measured from the apparent surfaces. However, this definition is misleading. The available free volume of the pore is much less than what would be suggested by this measure as the walls are built by placing spheres of “solid”; hence, the center of the fluid spheres will not be able to explore the extent of the volume defined by the said diameter. To obtain a more realistic definition of the available volume, we arbitrarily specified an “effective” wall surface to be at a distance 0.1 nm (*σ*_solid_/2) inward from that defined by the plane of the centers of the particles in the wall (c.f. Figure 3). In order to obtain the density of the adsorption region, the number of particles inside the cylindrical pore was counted over the time, and the average number of particles was taken when the loading in the pore was equilibrated. In the bulk region, the average number of particles was taken in the cubic zone showed in Figure 4. For the adsorption isotherms, the results are presented as the number of particles adsorbed as a function of the pressure in the system. In the simulation cell, the adsorbed region inside the cylinder was in equilibrium with the bulk phase, so the normal pressure inside the pores corresponded to the bulk (isotropic) pressure. Separate *NVT* isotropic single-phase simulations employing 500 particles were carried out at the density obtained from the bulk region to calculate the pressure of the system employing the classical virial (mechanical) route [38].

To compute the self-diffusion coefficient, *D*_z_, in the direction of the pore axis (*z* coordinate in Figure 4) we employed the Einstein relation, i.e., we followed the limiting value of the time dependence of the mean-square displacement (MSD) [17]:

(4) $$D_{z}=\frac{1}{2N}\underset{\Delta t\to \infty }{\lim}\frac{1}{\Delta t}\langle \sum_{i=1}^{N}{\left(z_{i}(t+\Delta t)-z_{i}(t)\right)}^{2}\rangle$$

where *N* is the number of molecules, and *z*(*t* + *∆t*) and *z*(*t*) are the coordinates of the particle at times *t* + *∆t* and *t*, respectively. To compute diffusion coefficients, molecular dynamics simulations in the microcanonical ensemble (*NVE*) were used for the fluid particles, while the solid particles forming the wall were restrained using harmonic potentials and held at a constant temperature (150 K) with a Nosé–Hoover thermostat. This method allowed the fluid particles, in contact with the position-restrained wall particles, to reach the desired temperature without being subject to a thermostat affecting the dynamics of the fluid particles. The simulation set-up consisted of an infinite cylinder where the fluid particles could move freely inside. The system was allowed to equilibrate, after which the MSD was calculated from Equation (4).

## 3. Results

### 3.1. Adsorption of Trimers in Cylindrical Pores

**Figure 5:**
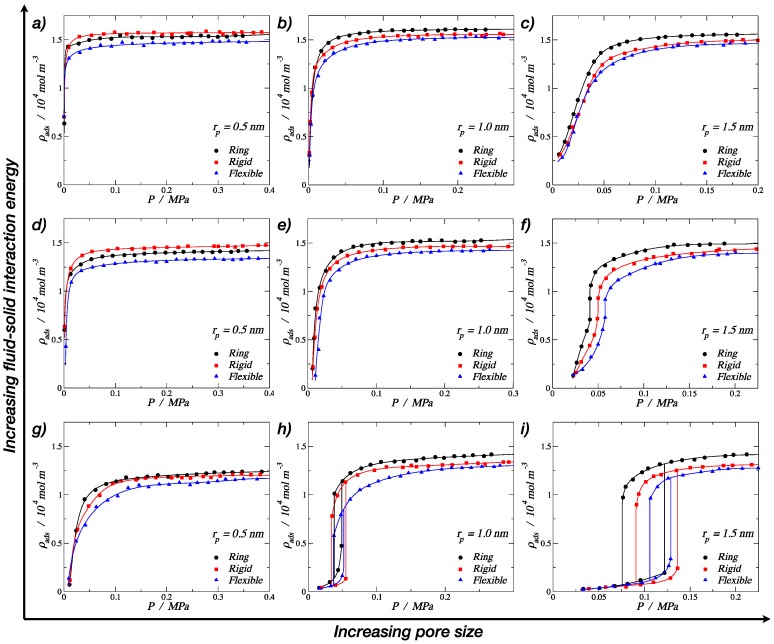
Adsorption isotherms at 150 K for trimers in a cylindrical pore. The top row corresponds to a strong fluid–solid energy interaction of *ε*p/*k*_b_ = 25.0 K per particle: (**a**) *r*p = 0.5 nm, (**b**) *r*p = 1.0 nm, and (**c**) *r*p = 1.5 nm. The middle row corresponds to the medium fluid–solid energy interaction of *ε*p/*k*_b_ = 19.3 K per particle: (**d**) *r*p = 0.5 nm, (**e**) *r*p = 1.0 nm, and (**f**) *r*p = 1.5 nm. The lower row corresponds to the weak fluid–solid energy interaction of *ε*p/*k*_b_ = 13.8 K per particle: (**g**) *r*p = 0.5 nm, (**h**) *r*p = 1.0 nm, and (**i**) *r*p = 1.5 nm. In all cases, the symbols in the isotherms correspond to rings (filled black circles), rigid linear chains (filled red squares), and fully flexible chains (filled blue triangles). Solid lines are empirical nonlinear fits. Error bars are of the size of the symbols.

The results obtained from molecular dynamics simulations for the adsorption of different trimer morphologies on cylindrical nanopores at 150 K are shown in this section. Figure 5 displays nine plots of adsorbed fluid density against the pressure of the bulk vapor, where each one corresponds to a specific pore radius and fluid–solid interaction energy, showing three curves corresponding to the three morphologies (ring-like, rigid linear, and fully-flexible) for different pore radii. The solid lines in each graph were obtained by fitting the MD results using nonlinear equations in order to show the trend of the isotherms. From Figure 5a–c, it can be observed that, for the pore radius 1.5 and 1.0 nm, the adsorption of trimers in cylindrical nanopores followed the following adsorption hierarchy: ring > rigid linear > fully flexible. This trend agreed with the bulk-phase behavior, where the ring-like configuration reached a higher density compared to the chain configurations (rigid linear and fully flexible). For the smallest pore radius (Figure 5a), an inversion of the adsorption hierarchy can be observed: rigid linear > ring > fully flexible. Visually, it can be seen that, for this small pore, the stiff trimers accommodated better along the axis of the pore, reaching a higher packing fraction.

From Figure 5a–c, it is also possible to see that all the adsorption curves accommodated to type I isotherms, according to the IUPAC classification [6]. Type I isotherms were, in general, observed for microporous solids with relatively small external surfaces, as shown in Figure 5a,b, and they can be subclassified as a type Ia, which is characteristic of narrow pores. Figure 5c shows a type Ib behavior, which is characteristic of slightly wider micropores [6]. The pore of rp = 1.5 nm is considered a mesopore, but the high fluid–solid energy interaction facilitated the molecular loading of trimers at lower pressures, inducing a type I behavior.

The last group of results, shown in the lower row of Figure 5g–i, correspond to the weaker fluid–solid energy interaction, with an *ε*p/*k*_b_ value of 13.8 K, which is around 70% of the medium energy and 55% of the strong energy interaction. In this case, the energy was relatively low, so it is not possible to observe the inversion in the adsorption hierarchy. Here, the rigid linear morphology showed the maximum density value for the smallest pore, but the ring-like and the rigid linear converged to similar values. A more important observation is that, with the lowest energy value, we were able to produce type V isotherms (Figure 5h,i), which is characteristic of macroporous solids with weak fluid–solid interactions. This weakness caused the uptake of fluid at low pressures to be quite small, but once the particles became adsorbed, the fluid–fluid interactions added to the fluid–solid interaction in order to adsorb further molecules. In the isotherms, this led to a first-order transition from vapor phase to liquid phase, which is reflected as an abrupt discontinuity in the curve.

In Figure 5h,i, hysteresis loops can be observed for all the three morphologies. Hysteresis in adsorption can be attributed to metastability and/or network effects, but for cylindrical pores, a delayed condensation is the result of metastability of the adsorbed multilayer [6]. The hysteresis loops observed here follow a type H1 (according to the IUPAC classification [6]), where network effects are minimal and the steep; narrow loop is a clear sign of delayed condensation on the adsorption branch. For the medium pore size, *r*p = 1.0 nm, narrower hysteresis loops were observed, and the condensation occurred at lower pressures. Again, for the smallest pore size, a type I behavior was observed but with a much lower density for the high-pressure liquid phase.

### 3.2. Nematic Order Parameter

The previous adsorption results showcased how the confinement effect changes the fluid phase behavior; for instance, within the smaller pores, the liquid densities of the rigid linear and the fully flexible chain showed different limiting values. Intriguingly, for the smallest pore size, the density that the rigid linear molecules could reach was much higher than for the corresponding fully flexible molecule and higher than the ring-like molecule for high fluid–solid interaction energy values.

The case of the rigid straight chains is unique as it is possible to observe a preferential order of those particles in the system. The order of the system can be monitored through an orientational order parameter, which is obtained from the average value of the second-order Legendre’s polynomial [39]:

(5) $$S=\langle \frac{3\cos^{2}\theta -1}{2}\rangle$$

where *θ* is the angle between the molecular axis and the local director, which corresponds to the preferred direction in a volume element of a liquid crystal sample. The local director vector here corresponds to the z-axis, which is the horizontal direction in Figure 4.

**Figure 6:**
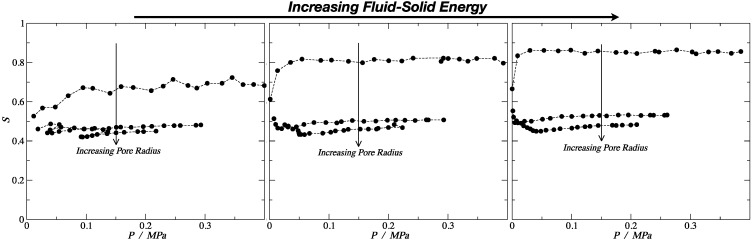
Nematic order parameter, *S*, as a function of the system pressure for the rigid linear morphology. From left to right, the energy of the fluid–solid interaction is increasing. Error bars are of the size of the symbols.

The order parameter lies within the interval 0 ≤ *S* ≤ 1, where the value of *S* = 1 indicates perfect ordering (all the molecules are ordered along the local director vector), and S = 0 corresponds to the situation of maximum symmetry of the isotropic phase with no order. The results presented in Figure 6 demonstrate that, for the smallest pore (dp ≈ 3*σ*), the order of the rigid (stiff) linear chains was significantly larger than for the other pore sizes. From both Figure 6 and Table 2, we can observe that, for the medium and strong fluid–solid interaction energies, the nematic order parameter, *S*, had a value above 0.8, which implied the presence of a nematic fluid, aligned with the pore axis. As a comparison, the nematic order parameter seen for flexible molecules (defining the molecular axis as the vector between the centers of the first and last bead) and the ring molecules (where the molecular axis is defined perpendicular to the plane of the beads) was roughly 0.3, independent of the confinement level and/or wall strength. In finite-size systems, one would expect nonzero values of the order parameters, even in the absence of ordering.

In fact, for a typical liquid crystal, the nematic order parameter *S* went from 0.3 to 0.8, and in the smallest pore for the medium and strong energy, the order parameter was higher than the typical maximum value, *S* = 0.815 and *S* = 0.86, respectively. This explains the anomalous adsorption hierarchy inversion discussed previously, where the rigid linear morphology showed a higher density than the fully flexible and the ring-like configuration. In the case of the weak interaction, the order parameter was *S* = 0.69, which was lower compared to the other two. This explains why the hierarchy inversion was not observed in this case. For the other two pore sizes, the values were between *S* = 0.45 and *S* = 0.53, implying some ordering in the system, but not enough to impose an enhanced packing. Moreover, for the fully flexible morphology, the order parameter was around *S* = 0.45; hence, the rigid linear and the fully flexible chains behaved in a similar way for bigger pore sizes and for the bulk phase.

### 3.3. Diffusion Coefficients

**Figure 7:**
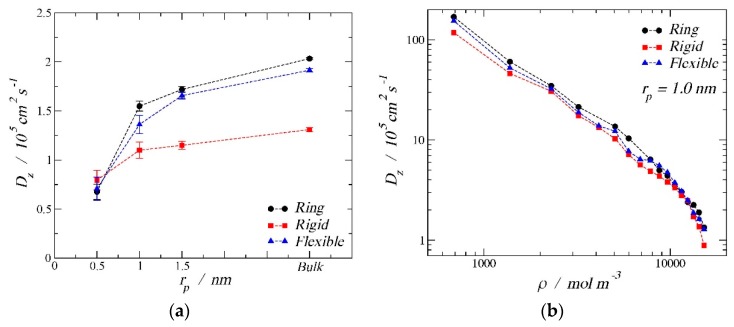
Self-diffusion coefficient in the *z* direction, *D*z, for the three morphologies at 150 K in a cylindrical nanopore. (**a**) The image on the left shows the diffusion coefficient as a function of the pore radius: 0.5 nm, 1.0 nm, 1.5 nm, and bulk phase at the high-density limit (15,167 mol/m^3^). (**b**)The image on the right shows diffusion coefficient as a function of the pore density for *r*p = 1.0 nm.

Besides the adsorption results from the previous section, an analysis of the diffusion coefficients is presented here to provide a better understanding of the confined phenomena. Figure 7a shows the self-diffusion coefficient in the z direction of the cylindrical nanopore as a function of the pore size for the three different morphologies at a high-density condition. Following the same nomenclature used in the adsorption section, black solid circles correspond to the ring-like molecules, red solid squares are the rigid linear molecules, and blue solid triangles represent the results for the fully flexible chains. In this graph, three different pore radii are shown:0.5 nm, 1.0 nm, and 1.5 nm. For comparison, we have included the bulk phase results (1/3 of the bulk three-dimensional diffusion coefficient) and plotted them arbitrarily at a radius of 3 nm to show the limiting trend. In all cases studied, confinement hindered the diffusion. It is possible to observe that, as the pore size increased, the self-diffusion coefficient in the z direction increased. The ring-like configuration had the largest self-diffusion coefficient for the bulk phase and for the two biggest pore sizes. The second largest diffusion coefficient was for the fully flexible chain, while the lowest coefficient was for the rigid linear morphology. An inversion can be observed for the smallest pore size, where the diffusion hierarchy followed the trend rigid linear > fully flexible > ring-like. By changing the energy of the fluid–solid interaction, the same trend can be observed with similar values of self-diffusion coefficient; therefore, only the so-called strong interaction is shown.

Figure 7b displays the self-diffusion coefficient in the z direction as a function of the density inside the pore. From this second graph, we can observe how, as expected, the diffusion coefficient decreased as the pore density increased, with similar values for the three different molecular configurations. Despite the similarities in the absolute values, one can consistently observes that the ring-like morphology had the highest diffusion coefficient, presumably to the larger sphericity of the ring configuration, which will aid with the diffusion through the fluid.

## 4. Conclusions

The results shed light on the relevance of molecular shape on the adsorption and transport properties of fluids in cylindrical nanopores. Although the phase equilibria and bulk fluid properties of linear and ring-like molecules were expected to be roughly similar, the results for adsorption and diffusion showed substantial differences.

The simulation results for adsorption showed that the molecular morphology has an important effect on the adsorption isotherm shape. Different types of isotherms were obtained by changing the size of the pore radius and the fluid–solid energy interactions. For the weakest fluid–solid energy, the adsorption isotherms presented hysteresis loops, an evidence of metastable phases in the adsorbed region. It was seen that the ring-like fluids exhibited a larger adsorption than the rigid linear models, and these latter ones adsorbed more than the flexible molecules, i.e., there was an adsorption hierarchy of ring-like > rigid linear > fully flexible. This generalization is in agreement with simulations of atomistic models of fluids in strong confinement [13]. There was an anomaly in the smallest pore, where the linear rigid molecules exhibited a higher adsorption. The analysis of the nematic order parameter in confinement confirmed that, for the rigid linear configuration and for the smallest pore, the system could reach a high positional order inside the cylinder. This enhanced packing, typical of a liquid crystal phase, enhanced its adsorption in the smallest pore and thus explained the anomalous trend in the adsorption behavior.

The diffusion (in the direction of the axis or *z*-direction) provided another important element to evaluate the presumed permeability of the models through nanoporous membranes. The widely accepted solution-diffusion model [40] suggests that permeation will be proportional to both the adsorption and the diffusion in the confined media. From the simulation results, the ring-like configuration had the highest self-diffusion coefficient for the bulk phase and for the pore radius of 1.0 nm and 1.5 nm. However, the difference with the straight analogue (the flexible chain) was well within the statistical error, and it would be inaccurate to suggest that there was a statistically significant difference amongst them. This, in conjunction with somehow similar adsorption isotherms presented by the ring and the flexible chains, suggests that the permeability of more globular molecules (e.g., a small aromatic) will be similar to that of a more linear one (e.g., an alkane) if and only if the solid–fluid interactions are similar in nature. On the other hand, if there is a significant difference in the way the fluid molecules interact with the surfaces, the adsorption isotherms will be different, and a selectivity induced by this effect is likely. An exception to this is again the case of the rigid linear molecules in the ultraconfined 1 nm pore. Particularly, in this case, due to the fluid behaving as a nematic liquid aligned with the pore axis, an abnormally high diffusion coefficient was observed. Given that the adsorption was also enhanced in this particular case, we would predict an enhanced permeability. We note that only in this particular latter case was the radius of gyration of the molecule commensurate with the pore radius. It is in these cases that a unique steric hindrance effect comes into play. Otherwise, it seems that the differences in molecular shape only modestly affect the adsorption and diffusion behavior.

## Figures and Tables

**Table 1:** Summary of the molecular parameters for the solid–fluid and fluid–fluid potentials. *k*_b_ is the Boltzmann constant.

	σ [nm]	*ε*/*k*_b_ [K]	λ_r_	λ_a_
C_cylinder,1_-LJ_fluid_	0.25	25.00	11.0	4.0
C_cylinder,2_-LJ_fluid_	0.25	19.30	11.0	4.0
C_cylinder,3_-LJ_fluid_	0.25	13.80	11.0	4.0
LJ_fluid_	0.30	100.00	12.0	6.0

**Table 2:** Nematic order parameter, *S*, for the adsorbed liquid phase for different pore sizes (*r*_p_) and different values of fluid–solid energy interaction (*ε_fluid-wall_*/*k*_b_). Values are averages taken at high densities/pressures.

*ε_fluid-wall_*/*k*_b_[K]	13.80			19.30			25.00		
*r*_p_ [nm]	0.5	1.0	1.5	0.5	1.0	1.5	0.5	1.0	1.5
*S*	0.69	0.477	0.45	0.815	0.506	0.47	0.86	0.53	0.48
